# Breast Cancer Screening Awareness, Knowledge, and Practice among Arab Women in the United Arab Emirates: A Cross-Sectional Survey

**DOI:** 10.1371/journal.pone.0105783

**Published:** 2014-09-29

**Authors:** Yusra E. Elobaid, Tar Ching Aw, Michal Grivna, Nico Nagelkerke

**Affiliations:** Institute of Public Health, College of Medicine & Health Sciences, United Arab Emirates University, Al Ain, United Arab Emirates; University of Tennessee, United States of America

## Abstract

**Background:**

Breast cancer screening can reduce morbidity and mortality and improve the survival rate for this malignancy. Low participation in screening programs has been attributable to many factors including lack of knowledge. The aim of this study was to assess breast cancer screening knowledge, attitudes and practices among women of screening age (≥40 years old) in the city of Al Ain, United Arab Emirates (UAE).

**Methods:**

A cross-sectional survey was conducted in 2013 using the Breast Cancer Awareness Measure (CAM). Four out of twelve cultural and religious community centers in Al Ain city were randomly selected. Two hundred and forty seven women were interviewed. Chi Square test and regression analysis were used to analyze the data.

**Results:**

Despite the increase in the uptake of screening modalities in our study group, a lack of knowledge about breast cancer screening is still evident. Almost half (44.8%) of women who never had a Clinical Breast Exam (CBE) and 44.1% of women who never had a mammography expressed a lack of knowledge about the existence of these screening techniques. Nearly one third of the participants interpreted the presence of a breast lump incorrectly and, moreover, expressed fewer worries about the nature of the lump than would normally be expected.

**Conclusions:**

The National screening program needs to be improved and directed towards more efficient and targeted utilization of resources. Healthcare professionals play a major role in alerting women to the importance of periodic screening.

## Introduction

Breast cancer is the most common cancer among women. The most recent estimate indicated that more than 1.6 million new cases of breast cancer occurred among women worldwide in 2010 [1]. Control of modifiable breast cancer risk factors such as maintaining a healthy weight, regular exercise and reducing alcohol intake could eventually have an impact in reducing the incidence of breast cancer. However, these strategies cannot eliminate the majority of breast cancers. Therefore, early detection in order to improve breast cancer outcome and survival remains the cornerstone of breast cancer control [2].

Breast cancer screening is one way of reducing morbidity and mortality and improving the survival rate [3]. In the United Arab Emirates (UAE), the Federal Ministry of Health initiated a breast cancer screening program in 1995. The program follows international guidelines using a combination of monthly Breast Self-Exam (BSE), regular Clinical Breast Exam (CBE), and a mammography every two years after the age of 40 [4]–[5]. UAE society has experienced major shifts in lifestyle mainly because of acquired oil wealth since the formation of the country in 1971 [6]. Screening services are widely available and free of charge for UAE national women over the age of forty. Despite this, the participation of women in screening activities is very low (10%), with more than 65% of breast cancer patients presented at advanced stages [7]. Many barriers to breast cancer screening and underutilization of services have been studied worldwide, but in the UAE, a country with specific cultural, traditional and religious values, very few studies have been conducted. A previous study reported poor knowledge of breast cancer among women in Al Ain, UAE [6]. No other studies have been conducted to assess the impact of the national screening program. We therefore aimed to assess the breast cancer screening awareness, knowledge and practice among women of screening age in the city of Al Ain, UAE.

## Methods

A cross-sectional survey was conducted in 2013. The sample for the survey was selected from community, cultural and religious centers in Al Ain city. These centers are designed for women to socialize and they additionally provide an organized forum for religious gatherings. Four centers from a total of 12 were randomly selected. Management were approached and study days were selected. Each center was visited once. Every woman aged 40 years and above attending the center during the study day was approached and invited to participate. The women who agreed to participate were interviewed and answered a prepared questionnaire. A modified Arabic version of the Cancer Awareness Measure (CAM) version 2.1 [8] was used. To accommodate local and national cultural differences, several modifications were made and a few questions were added regarding the women’s interpretation of breast lumps. The questionnaire was divided into three sections; the first covered the socio-demographic characteristics (age, nationality, education level, age at menarche, breastfeeding practice and contraception methods). The second section included questions relating to breast cancer knowledge, risk factors and warning signs. Correct answers scored 1, false answers or ‘don’t knows’ scored 0. The maximum attainable score was 16. Knowledge scores were then grouped into four categories; good, moderate, poor and very poor. The third section covered the knowledge and practice of regular BSE, CBE and mammography. In the general knowledge section of the questionnaire, the women were asked if they would seek medical attention if they noticed a lump in their breast. Questionnaires were marked based on the Cancer Awareness Measure (CAM) version 2.1 answer sheet.

Using statistical package for the social sciences (IBM-SPSS version 19.1, Chicago, II, USA), data were analyzed by regrouping, frequencies, and cross-tabulations. Logistic regression was used to identify predictors of screening. A p value < 0.05 was considered significant.

Al Ain District Ethics Committee and the United Arab Emirates University approved this study (Protocol No. CRD 260/13).

## Statistical Analysis

### Results

The research was undertaken in March, 2013. A total of 305 eligible women were approached. Two hundred and forty seven agreed to participate (response rate 81%). UAE nationals constituted 43.3% (n = 107), the remainder being citizens of other Arab countries. The majority were married (88.7%, n = 219). Around 94% (n = 233) were literate. Mean age was 46.1 years (± SD 4.95) (Table 1). The screening uptake in our study group was BSE 48.6% (n = 120), CBE 49.4% (n = 122), and mammography 44.9% (n = 111). In a total of 120 women who did not regularly perform BSE, 34.1% (n = 41) had not previously heard of BSE. Almost 28% (n = 33) expressed a willingness to perform BSE, if advised of proper procedures. However, 38.3% (n = 46) claimed good knowledge of BSE procedures but did not perform them due to fear of finding something.

**Table 1:** Socio-demographic variables of women who attend breast cancer screening (BSE, CBE, mammography) n = 247.

Category	Sub-category	Total n (%)	BSE n (%)	CBE n (%)	Mammography n (%)
Nationality	UAE national	107 (43.3)	56 (52.3)	59 (55.1)	53 (50%)
	Non-UAE national	140 (56.7)	71 (50.7)	63 (45)	58 (41.4)
Age	40–49 years	200 (81)	101 (50.5)	95 (47.5)	81 (40.7)
	≥50 years	47 (19)	26 (55.3)	27 (57.4)	30 (63.8)
Education	No formal education	14 (5.6)	8 (57.1)	9 (64.3)	11 (78.6)
	School level	115 (46.5)	55 (48.2)	58 (50.9)	52 (46)
	College/university level	118 (47.9)	64 (54.2)	54 (45.8)	47 (39.8)
Total		247 (100)	127 (51.4)	122 (49.4)	111 (45.1)

BSE: breast self-exam.

CBE: clinical breast exam.

Additionally, 22% (n = 54) stated that they were unsure if breast cancer was contagious, 38% (n = 93) believed that only women got breast cancer, and 43% (n = 106) believed that breast cancer was the most common type of cancer in women. Interestingly, 26% (n = 64) believed that breastfeeding was protective against breast cancer. Nearly 17% (n = 42) thought that a mammogram was done only upon symptomatic complaints, and 16% (n = 39) were not sure when to go for mammography. It was discovered that only 5% (n = 12) had a good general knowledge of breast cancer while 14.6% (n = 36) had a very poor knowledge. UAE national women scored better than non-UAE national women (means 7.14 vs. 6.99) (Table 2). Generally, younger women (40–49) had better scores regarding knowledge than older women (>49). The level of education was positively associated with better knowledge scores (p = <0.001). Participation in mammography was negatively associated with education level: OR was 0.18 and 0.13 for school level and university level respectively, compared to women with no formal education. Almost 32% (n = 23) who held a university degree and did not perform regular mammography believed that it was not appropriate for their age group. Nearly 45% (n = 56) who never had CBE and 44% (n = 60) who never had mammography expressed a lack of knowledge about the very existence of these screening techniques. The study suggests that breast cancer knowledge among the target group is inadequate.

**Table 2:** General knowledge score by nationality, age and education.

Category	Sub-category	Frequency n (%)	General knowledge score Mean (SD)
Nationality	UAE nationals	107 (43.3)	7.14 (2.87)
	Non-UAE nationals	140 (56.7)	6.99 (3.29)
Age group	40–49	200 (81)	7.12 (3.18)
	>49	47 (19)	6.78 (2.83)
Highest level of education	No formal education	14 (5.6)	5.57 (2.53)
	School level	115 (46.5)	6.56 (2.97)
	College or University	118 (47.9)	7.71 (3.18)

Non-UAE nationals had a poorer mammography uptake compared to UAE nationals, but the association was not significant (p = 0.75). UAE nationals reported being given breast health counseling more than non-UAE nationals (52.8%; n = 57 vs. 47.2%; n = 66) (p = 0.002). In the study, 81% (n = 103), 41.3% (50) and 82.6% (n = 92) of women who practiced BSE, CBE and mammography respectively, stated that it was recommended by their healthcare provider. 63% (n = 85) of the women who reported not having had a mammogram had never been given breast health counseling (P = <0.001). Some women who never had CBE or mammography misunderstood that they were not in the target group for screening and they believed it was for older women (41.6% and 33.8% in CBE and mammography respectively). Older women (≥49 years of age) were more likely to go for a mammography p = <0.001 (OR 1.14, CI 95% 1.07–1.22) (Table 3). Reasons for not regularly performing BSE, CBE or mammography by women are outlined in (Table 4).

**Table 3:** Predictors of breast cancer screening.

Variable/predictor	BSE		CBE		Mammography	
	OR (95%CI)	p-value	OR (95%CI)	p-value	OR (95%CI)	p-value
Age	0.96 (0.90–1.02)	0.24	0.94 (0.88–1.00)	0.06	1.14 (1.07–1.22)	<0.001
Education						
School level*	2.59 (0.59–11.24)	0.20	2.61 (0.60–11.22)	0.19	0.18 (0.37–0.91)	0.03
University level*	1.79 (0.39–8.13)	0.44	3.52 (0.78–15.90)	0.10	0.13 (0.02–0.72)	0.01
Knowledge score	0.81 (0.73–0.90)	<0.001	0.83 (0.75–0.92)	0.001	1.20 (1.09–1.33)	<0.001
Breast health counseling	4.30 (2.36–7.85)	<0.001	3.95 (2.21–7.05)	<0.001	0.38(0.21–0.69)	0.001

*As compared to no formal education group.

**Table 4:** Reasons for not performing regular BSE, CBE, and mammography.

Screening method	Reason	n (%)	Nationality		Education		
			UAE-Nationaln (%)	Non UAE- Nationaln (%)	No formal educationn (%)	Schooln (%)	Universityn (%)
BSE	I would like to do it but I do not know how	33 (27.5)	12 (23.5)	21 (30.4)	4 (66.7)	20 (33.3)	9 (16.7)
	I know about it but I am afraid to do it orhave no time to do it	46 (38.3)	17 (33.3)	29 (42)	1 (16.7)	15 (25)	30 (55.6)
	Never heard of it	41 (34.2)	22 (43.1)	19 (27.5)	1 (16.7)	25 (41.7)	15 (27.8)
Total		120 (100)	51 (42.5)	69 (57.5)	6 (5)	60 (50)	54 (45)
CBE	Not for my age group	52 (41.6)	18 (36.7)	34 (44.7)	2 (40)	27 (47.4)	23 (36.5)
	I know about it but I am afraid to do it orhave no time to do it	17 (13.6)	8 (16.3)	9 (11.8)	0 (0)	5 (8.8)	12 (19)
	Never heard of it	56 (44.8)	23 (46.9)	33 (43.4)	3 (60)	25 (43.9)	28 (44.4)
Total		125 (100)	49 (39.2)	76 (60.8)	5 (4)	57 (45.6)	63 (50.4)
Mammography	Not for my age group	46 (33.8)	14 (25.9)	32 (39)	2 (66.7)	21 (33.9)	23 (32.4)
	I know about it but I am afraid to do it orhave no time to do it	30 (22)	14 (25.9)	16 (19.5)	0 (0)	13 (21)	17 (23.9)
	Never heard of it	60 (44.2)	26 (48.1)	34 (41.5)	1 (33.3)	28 (45.2)	31 (43.7)
Total		136 (100)	54 (39.7)	82 (60.3)	3 (2.2)	62 (45.6)	71 (52.2)

Nearly a third of the women interpreted a breast lump incorrectly, expressing fewer worries about the nature of the lump. They indicated no intention to seek medical attention. They perceived the lump to be due to normal hormonal changes (affecting women at menopausal age) or to breastfeeding. The correct interpretation of a breast lump was positively associated with general knowledge p = <0.001 (OR 1.26 CI 95% 1.12–1.41) and with the presence of family history p = 0.21 (OR 1.74 CI 95% 0.71–4.22).

### Discussion

The results of the study showed that there remains a lack of awareness about breast cancer screening and a consequent underutilization of screening services. It also highlighted some misconceptions regarding the timely medical advice upon the discovery of a breast lump.

The results are consistent with those of a Saudi Arabian study [9] in which screening services were noted to be underutilized and mainly used for the diagnosis of breast lesions.

Screening services are widely available under health insurance in the UAE. Indeed, free screening is offered by some facilities. Regrettably, women are not referred for screening in these facilities. In general, Arab women regard physicians’ offices, clinics and other healthcare facilities as places to go only when one feels ill or when symptoms appear [9]. Moreover, 38% (n = 94) of the women gained breast cancer knowledge from their healthcare provider. Almost 18% (n = 44) believed that a mammogram is only done upon symptoms such as pain or inflammation. Consequently, this perception serves as a barrier to health promotion efforts to achieve high participation rates. This has implications on the effectiveness and success of current screening programs.

The findings showed a significant improvement in the screening uptake compared with findings of a similar study conducted in in Al Ain [6], which showed uptake of (12.7%), (13.8%), (10.3%) for BSE, CBE and mammography respectively. The screening uptake was also higher than in Qatar and Saudi Arabia [10]–[11]. One of the major barriers to screening is the lack of knowledge about the benefits of early detection [5]. Studies have also implied that with improved knowledge, the screening uptake will increase [12]–[13].

### Breast Self-Exam (BSE)

In contrast to the study conducted previously [6], older women (≥49 years of age) were found to practice BSE more frequently than younger age group (40–49 years of age). In our study, 81.6% of women practicing BSE were given instructions by a healthcare provider compared to 38% in the previous study [6]. This might be due to the increased early detection and awareness activities conducted by the local health authorities during the past decade [7]. Thus, offering information to women on how to perform BSE improves screening practices [14]. After introducing the national screening program in the UK in 1980s, rates of advanced breast cancer reduced dramatically [15]. In the UAE, the efforts of Health Authority Abu Dhabi are improving and resources are directed toward better implementation of screening activities [7]. The improved rate of BSE practice in this study population indicates the success of the educational and awareness programs.

### Clinical Breast Exam (CBE) and/or Mammography

In our study only 58% of women reported having a history of CBE or a mammography in the past few years. This is low compared to other countries, for example 72% of the target population in Canada reported to have had a mammogram in the past two years [16]. In the UK more than 80% of women aged 50–69 are reported to have had mammography in the previous three years [17]. However, comparing the UAE experience to a neighboring country, Qatari women reported low participation rate in breast cancer screening activities, 23.3% and 22.5% for CBE and mammography respectively, despite having adequate knowledge level [10]. The literature from developed countries indicated that breast cancer risk increases with increased age at diagnosis [18]. However, the age at diagnosis of breast cancer in the Arab world was reported to be a decade earlier than in western countries [19]. Despite the fact that the recommended age to start mammography is ≥40, in our study older women (>49) tended to practice mammography more than younger women (40–49). Younger women felt they did not need breast cancer screening before the age of 50 and they are too young to develop breast cancer. Although the level of knowledge of breast cancer was higher in more educated women, they were not very keen in practicing mammography. This might be related to the fear of frequent radiation exposure from periodic mammography or the controversy of over-screening. Educated women might be motivated to practice BSE and CBE instead. Breast health counseling improved the uptake of BSE and CBE as during the visit to the healthcare provider women are examined and educated about the performance of BSE. Our findings showed that 80% of women who reported having a history of CBE and/or 83% mammography indicated that these screening techniques were recommended by their healthcare provider. Improvement in the uptake of CBE has been linked to the increased awareness about the importance of attending regular CBE.

Other misconception is believe, that mammography should be performed once and not in a regular bi-annual basis. Health care providers play a major role in delivering the message of the necessity of regular breast cancer screening. Earlier studies reported that physician recommendation was the single strongest predictor of breast cancer screening [20]–[23].

### Breast lump interpretation

Women easily fall in misconceptions and myths about breast lumps. Our study highlights the importance of misconceptions about breast lumps in contributing to longer delay. Nearly 57% (n = 141) of participants misunderstood screening as seeking medical attention only when having symptomatic breast complaint. Common barriers towards screening included fear of pain and embarrassment, fear of radiation causing cancer, and perceived inadequate facilities. Women would change their minds if they became symptomatic or if their doctors, family or friends encouraged them. Increased level of knowledge was present in women who did not interpret breast lump as threatening or alarming sign of breast cancer.

### Strengths and limitations

This cross sectional survey is one of the few community based studies on the effect of national screening program assessing knowledge and practice of breast cancer screening modalities among women in Al Ain city. We used a previously validated questionnaire [8]. Some limitations to this study should be considered. Since, this study was not completely based on a random sample, the findings may not be generalized to all women in the UAE. The inability to select a random sample from Al Ain community may have introduced selection bias. Moreover, women attending the community cultural and religious center might differ from those in the community. Not all women can attend these centers due to work or live constraints. Non-response bias might also be exemplified in this study with the assumption that non respondent women to the questionnaire might differ from women who were willing to respond. However, the high response rate (81%) has minimized the non-response bias. Mammography use and BSE performance were self-reported, whereas it can be more valid via medical record review.

### Conclusion

Despite the increase in the uptake of screening modalities in our study group, lack of knowledge about breast cancer screening is still evident. The positive association between knowledge of breast cancer and screening uptake is clear, but it is difficult to determine whether breast cancer knowledge preceded cancer screening or whether previous exposure to screening increased knowledge. A follow up case control study may help establish the direction of causality. Most efforts of local health authorities were targeting individual barriers of screening; more holistic approach to tackle socioeconomic, cultural, and religious factors is needed. In particular, it should be emphasized that screening should be performed even when asymptomatic. More awareness should also be raised towards available subsidies and facilities. Opportunistic screening adopted by the national screening program in UAE does not completely fulfill the program objectives, so efforts need to be directed toward organized, directed and better utilization of resources. Language and trust barriers between women and healthcare providers need to be minimized for practical and efficient implementation of screening programs. There is an urgent need for coordinated awareness campaigns organized by the local health authority and healthcare providers.

## Supporting Information

Data S1 (XLSX)
